# The risk and prognostic factors for G1 pancreatic neuroendocrine tumors: A retrospective analysis of the SEER database

**DOI:** 10.3389/fonc.2022.993524

**Published:** 2022-10-06

**Authors:** Zhengqi Wu, Xiaotong Qiu, Yao Zhi, Xiaoju Shi, Guoyue Lv

**Affiliations:** Department of Hepatobiliary and Pancreatic Surgery, General Surgery Center, First Hospital of Jilin University, Changchun, China

**Keywords:** cancer-specific survival, prognosis, competing risk, SEER, pancreatic neuroendocrine tumors

## Abstract

**Background:**

Pancreatic neuroendocrine tumors (pNETs) are rare neuroendocrine neoplasms (NENs) for which little is known about their clinical features, treatment options, and survival prognosis. The purpose of this study is to evaluate the risk factors affecting the overall survival (OS) and cancer-specific survival (CSS) in patients with grade 1 pNETs (G1 pNETs) and to provide a new theoretical basis for clinical diagnosis and treatment.

**Methods:**

A retrospective analysis of individuals with G1 pNETs registered in the Surveillance, Epidemiology, End Results (SEER) database was performed. Risk factors affecting OS and CSS were analyzed using Kaplan-Meier analysis, Cox proportional hazards model, and Fine-Gray competing-risk model.

**Results:**

A total of 751 patients were included, most of whom were white (77.2%) women (53.9%) under the age of 60 years (54.9%), of whom 66 died of pNETs (8.78%) and 34 died of other causes (4.52%). Patients who were older than 60 years at diagnosis (hazard ratio [HR] = 1.866, 95% confidence interval [CI]: 1.242-2.805) had worse OS. And stage in the regional extent (HR = 1.777, 95% CI: 1.006-3.137) or distance extent (HR = 4.540, 95% CI: 2.439-8.453) had worse OS. Patients who delayed treatment after diagnosis had shorter CSS (delayed treatment < 1 month: HR = 1.933, 95% CI: 0.863-4.333; delayed treatment ≥ 1 month: HR = 2.208; 95% CI:1.047-4.654). Patients with lymph node metastasis (HR = 1.989, 95% CI: 1.137-3.479) or distant metastasis (HR = 5.625, 95% CI: 1.892-16.726) had worse CSS. Acceptance of surgery can significantly improve the patient’s OS and CSS. OS (partial pancreatectomy [PP]: HR = 0.350, 95% CI: 0.182-0.672; pancreatectomy and duodenectomy [PD]: HR = 0.426, 95% CI: 0.222-0.815; total pancreatectomy [TP]: HR = 0.495, 95% CI: 0.193-1.267). CSS(PP: HR = 0.148, 95% CI: 0.0054-0.401; PD: HR = 0.332, 95% CI: 0.150-0.730; TP: HR = 0.69, 95% CI: 0.254-1.872).

**Conclusion:**

Age and stage were identified as independent risk factors for OS. Delayed treatment, N stage and M stage were independent risk factors for CSS. Only surgery was identified as independent protective factors for OS and CSS.

## 1. Introduction

Neuroendocrine neoplasms (NENs) are a type of tumors that has different clinical and biological characteristics, with the pancreas being common site of disease (1–4). The first classification of gastroenteropancreatic neuroendocrine tumors (NETs) proposed by The World Health Organization (WHO) in 1980 used the term “carcinoid” to describe most gastrointestinal NETs except islet cell tumors and small cell carcinomas (1). Currently, the 5th edition classification and grading standards released by WHO in 2019 are usually used to classify and grade the tissue differentiation degree and cell proliferation activity of pancreatic NENs (pNENs) (5). pNENs are divided into well-differentiated NETs and poorly-differentiated neuroendocrine carcinomas (NECs), in which NETs can be classified into grade 1, grade 2, and grade 3 (G1, G2, G3) based on mitotic counts and Ki-67 labeling index, and the invasiveness gradually increases from G1 to G3 grades, while NECs are not specifically graded, which are considered to be the most invasive (5, 6).

pNENs are rare pancreatic tumors, the incidence of which has increased significantly in recent years. With the advancement of inspection technology and the popularization of health examinations, the clinical detection rate of pNENs has also shown an upward trend (7, 8). More than half of the pNENs are G1 pancreatic NETs (G1 pNETs). Due to the indolent and less aggressive nature of G1 pNETs, the prognostic factors of G1 pNETs are easily neglected by clinical researchers. Moreover, the studies on G1 pNETs are only reflected in a few case reports or single centers, which makes the difference in the epidemiological and clinicopathological characteristics of G1 pNETs not well investigated based on large population databases. Therefore, in this study, we used the national-scale Surveillance, Epidemiology, End Results (SEER) database to expand the number of cases and to obtain clinical and survival information on patients with G1 pNETs. Kaplan-Meier analysis, Cox proportional hazards model and competing-risk model were used to analyze OS and CSS in patients with G1 pNETs, aiming to more comprehensively and accurately identify independent risk factors for OS and CSS. Ultimately, it can provide a new perspective for the clinical individualized treatment of G1 pNETs.

## 2. Materials and methods

### 2.1 Data retrieval

The SEER registry is a multicentric and reliable database for cancer research. This database collects data from 17 regions of the United States, covering approximately 28% of the total U.S. population. Information obtained from the SEER database includes patient demographics, primary cancer site, disease grade/stage, surgery, radiotherapy, chemotherapy and survival status.

### 2.2 Patient screening

We selected patients diagnosed with G1 pNETs from 2010 to 2015 in the SEER database according to the International Classification of Diseases (ICD) and the 5th edition histological codes (8240/3). Follow-up until November 2021. The inclusion criteria were as follows: a. A precise histopathological diagnosis of G1 pNETs; b. The primary site of the tumor is located in the pancreas with only one primary tumor; c. Follow-up for more than one month; d. People over 18 years old.

### 2.3 Covariate selection

Demographic variables include age, sex, race, marital status, household income, delayed treatment, tumor size, stage (SEER-specific), TNM (6th), the primary site of surgery, radiotherapy, chemotherapy, SEER classification of causes of death, months of survival, and final status. No privately identifiable information was obtained from the SEER database.

Age at diagnosis was classified into two groups: “< 60” and “≥ 60” years old. The race was recorded as white, black or others. Sex was described as male and female. Marital status was divided into married and unmarried (including single, separated, divorced and widowed). Household income was classified into two groups: “< 65,000$” and “≥ 65,000$”. Delayed treatment was divided into immediate treatment, delayed treatment within 1 month, and more than 1 month. Tumor size was assigned into two groups: “< 2 cm” and “≥ 2 cm”. TNM was assigned to “I”, “II”, “III”, and “IV”. Stage was classified into three subgroups: “localized”, “regional” and “distant”. Treatment included chemotherapy, radiotherapy and surgery. Radiotherapy or chemotherapy was divided into Yes or No. The surgery was assigned to the following four types: “None”(code 00), “partial pancreatectomy (PP)” (code 25,30), “pancreatectomy and duodenectomy (PD)” (codes 35-37), and “total pancreatectomy (TP)” (code 40).

### 2.4 Statistical analysis

Basic statistical analysis was performed on the demographic, clinical, and pathological characteristics of patients with G1 NETs. The primary endpoints of this study were OS and CSS in patients with G1 NETs. OS was defined as the time from diagnosis to death of any cause, while CSS was defined as the time from diagnosis to death of G1 NETs. The related variables affecting OS or CSS were selected by the log-rank test in the Kaplan-Meier analysis. Using the multivariate Cox proportional hazards model, the forward selection method included variables in the model to identify independent predictors of OS. Because the death of other causes is a competing event for cancer-specific death (CSD) in patients with G1 NETs, the use of Cox proportional hazards model may lead to overestimating the cumulative incidence of CSD. Therefore, the Fine-Gray competing-risk model was used to estimate the cumulative incidence of CSD by the “cmprsk” R package. Multivariate analysis was performed using the competing-risk model to identify independent risk factors affecting CSS. HR and 95% CI were measured to assess the strength of the association between each variable and survival. A two-sided *P*-value < 0.05 was used to indicate statistical significance.

## 3.Results

### 3.1 Clinical basic characteristics

**Figure 1:**
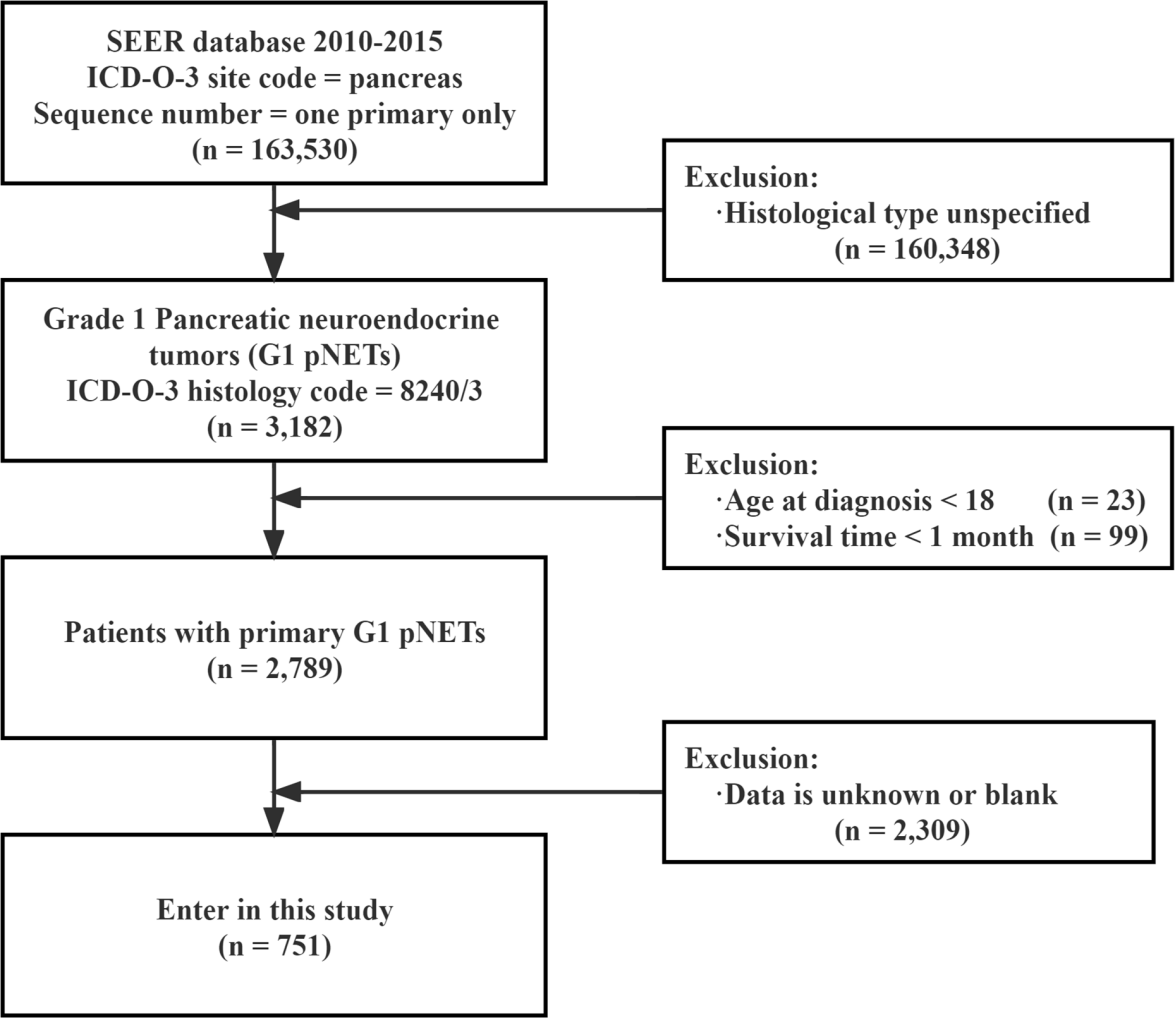
Flow chart showing the inclusion and exclusion process of patients in our study SEER : Surveillance, Epidemiology, End Results.

A total of 2,789 patients with G1 NETs were extracted from the SEER database. After selecting according to the inclusion criteria, there were 751 patients in the final cohort. The selection process is shown in **Figure 1**. Among them, 54.9% were younger than 60 years old at the time of diagnosis, 53.9% were female, 77.2% were white, 55.4% had household income not less than 65,000$, 66.7% were married, and 61.8% had tumor diameter less than 2 cm. In TNM, nearly half of patients (43.4%) were diagnosed at T1 stage. Most patients did not have regional lymph nodes (N0, 81.8%) or distant metastasis (M0, 87.1%). In terms of stage, the most common was localized extent (63.6%), followed by regional extent (22.1%). After the diagnosis was confirmed, 41.8% of the patients were treated immediately, while 25.8% were delayed for one month, and 32.4% were delayed for more than one month. Regarding treatment, 89.2% of the patients underwent surgery, of which 54.5% underwent PP; 28.1% underwent PD; and 6.7% underwent TP. Only a minority received radiotherapy (1.9%) and chemotherapy (10%). Detailed information is shown **Table 1**. At the last follow-up, 100 patients (13.3%) died, of which 66 (8.78%) died of G1 NETs and 34 (4.52%) died of other causes.

**Table 1:** Clinicopathological characteristics of patients with G1 pNETs.

Parameter	Subgroup	n (%)
Age	< 60	412 (54.9)
	≥ 60	339 (45.1)
Sex	Female	405 (53.9)
	Male	346 (46.1)
Race	White	580 (77.2)
	Black	85 (11.3)
	Other	86 (11.5)
Household income	< 65000	335 (44.6)
	≥ 65000	416 (55.4)
Marital status	Married	501 (66.7)
	Unmarried	250 (33.3)
Tumor size	< 2cm	464 (61.8)
	≥ 2cm	287 (38.2)
Delayed treatment	None	314 (41.8)
	< 1 month	194 (25.8)
	≥ 1 month	243 (32.4)
T stage	T1	326 (43.4)
	T2	230 (30.6)
	T3	172 (22.9)
	T4	23 (3.1)
N stage	N0	614 (81.8)
	N1	137 (18.2)
M stage	M0	654 (87.1)
	M1	97 (12.9)
TNM	I	478 (63.6)
	II	162 (21.6)
	III	14 (1.9)
	IV	97 (12.9)
Stage	Localized	478 (63.6)
	Regional	166 (22.1)
	Distant	107 (14.2)
Surgery	None	81 (10.8)
	PP	409 (54.5)
	PD	211 (28.1)
	TP	50 (6.7)
Radiotherapy	No	737 (98.1)
	Yes	14 (1.9)
Chemotherapy	No	676 (90.0)
	Yes	75 (10.0)

PP, Partial pancreatectomy; PD, Pancreatectomy and duodenectomy; TP, Total pancreatectomy; G1 pNETs, grade 1 Pancreatic neuroendocrine tumors.

### 3.2 Survival analysis

#### 3.2.1 Overall survival

**Figure 2:**
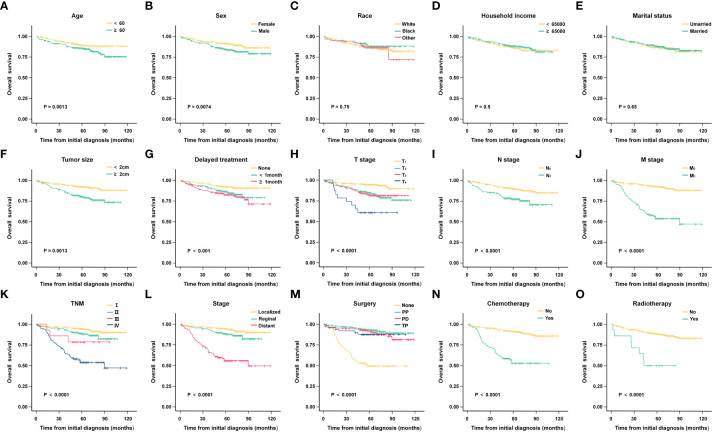
Kaplan-Meier analysis of overall survival (OS) in different subgroups. **(A)** Age; **(B)** Sex; **(C)** Race; **(D)** Household income; **(E)** Marital status; **(F)** Tumor size; **(G)** Delayed treatment; **(H)** T stage; **(I)** N stage; **(J)** M stage; **(K)** TNM; **(L)** Stage; **(M)**Surgery; **(N)** Chemotherapy; **(O)**Radiotherapy.

According to the Kaplan-Meier analysis and Log-rank test results, age (P = 0.017), sex (P = 0.007), tumor size (P < 0.001), delayed treatment (P < 0.001), TNM (P < 0.001), stage (P < 0.001), surgery (P < 0.001), chemotherapy (P < 0.001), and radiotherapy (P < 0.001) significantly affected the OS of patients. As for race (P = 0.751), household income (P = 0.500), and marital status (P = 0.650), none of them seemed to have a significant effect on OS in patients with G1 NETs. (The results are also visualized in **Figure 2**).

**Figure 3:**
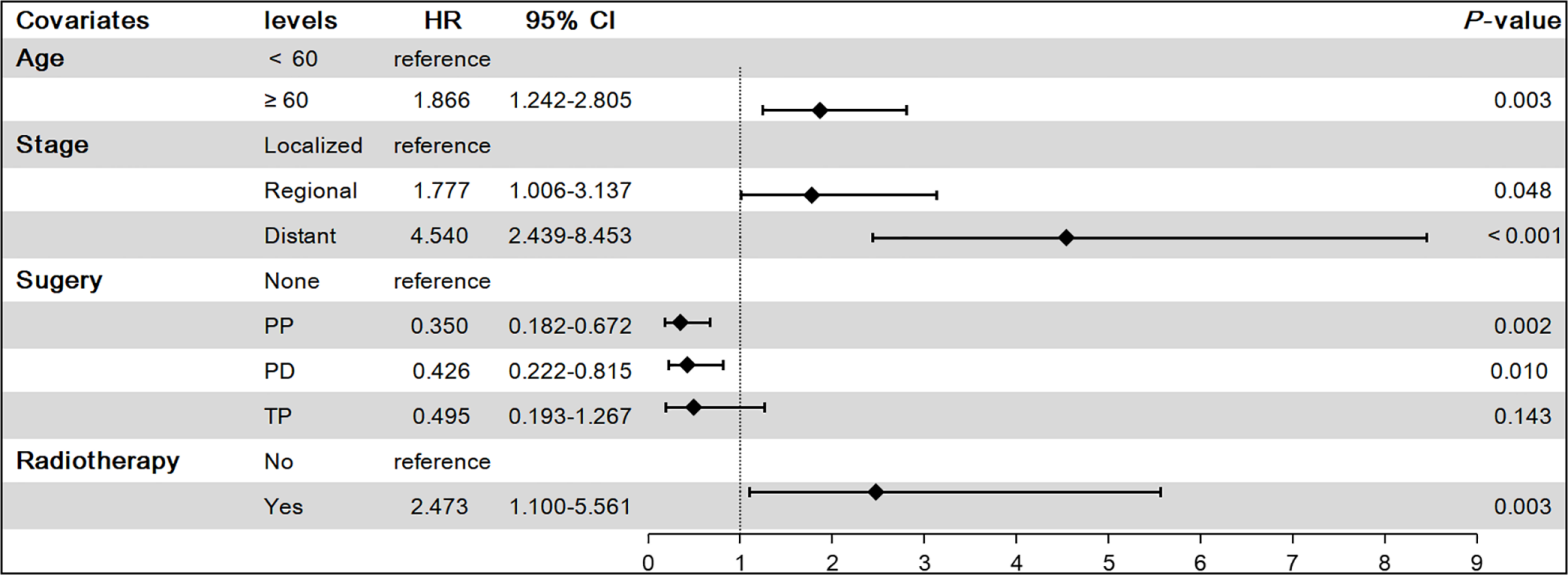
Results of multivariate Cox proportional hazards model on overall survival(OS). PP, Partial pancreatectomy; PD, Pancreatectomy and duodenectomy; TP, Total pancreatectomy.

To find independent influencing factors affecting OS in patients, we performed a multivariate Cox proportional hazards analysis for variables with *P*-values < 0.1 on univariate analysis. The results showed that age, stage, surgery and radiotherapy were independent factors affecting OS (**Figure 3**). Compared with patients aged younger than 60 years, patients older than 60 had shorter OS (HR = 1.866, 95% CI: 1.242-2.805). Patients with regional extent (HR = 1.777, 95% CI: 1.006-3.137) and distant extent (HR = 4.540, 95% CI: 2.439-8.453) had shorter OS relative to localized extent in SEER stage. Surgical treatment can significantly prolong the OS of patients, including PP (HR = 0.350, 95% CI: 0.182-0.672), PD (HR = 0.426, 95% CI: 0.222-0.815), TP (HR = 0.495, 95% CI: 0.193-1.267). Patients who received radiotherapy had worse OS (HR = 2.473, 95% CI: 1.100-5.561).

#### 3.2.2 Cancer-specific survival analysis

**Figure 4:**
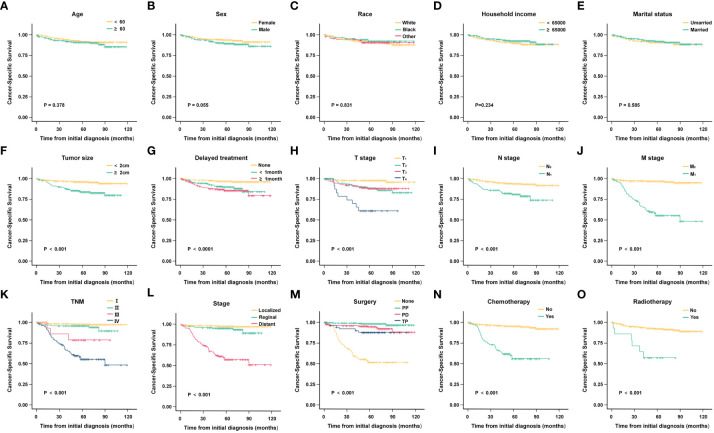
Kaplan-Meier analysis of cancer-specific survival (CSS) in different subgroups. **(A)** Age; **(B)** Sex; **(C)** Race; **(D)** Household income; **(E)** Marital status; **(F)** Tumor size; **(G)** Delayed treatment; **(H)** T stage; **(I)** N stage; **(J)** M stage; **(K)** TNM; **(L)** Stage; **(M)**Surgery; **(N)** Chemotherapy; **(O)** Radiotherapy.

**Figure 5:**
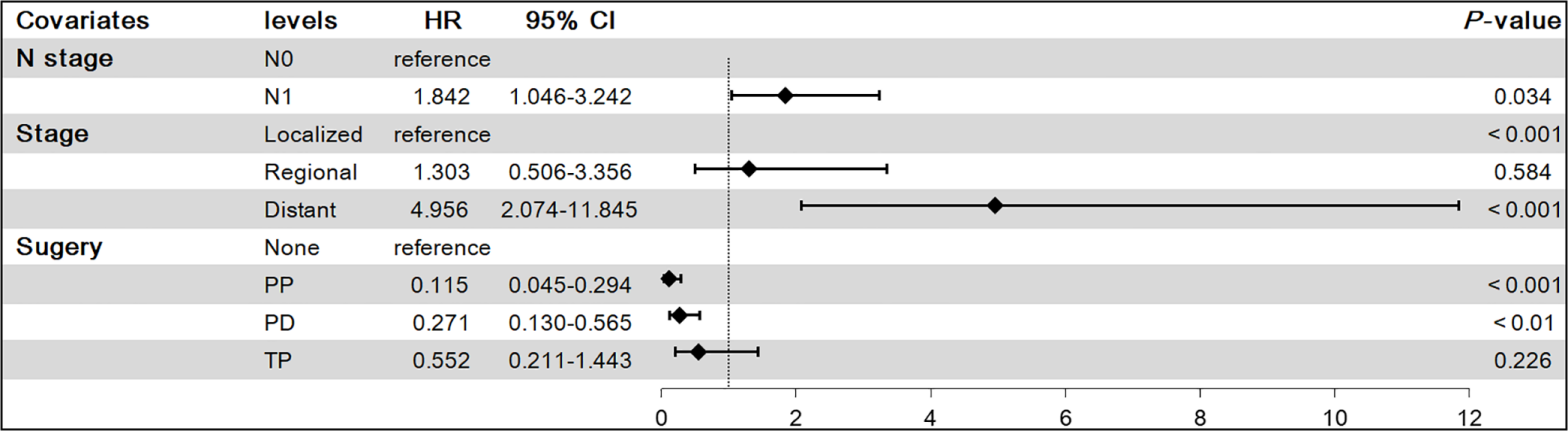
Results of multivariate Cox proportional hazards model on cancer-specific survival (CSS). PP, Partial pancreatectomy; PD, Pancreatectomy and duodenectomy; TP, Total pancreatectomy.

Kaplan-Meier analysis results indicated that tumor size (P < 0.001), delayed treatment (P < 0.001),TNM (P < 0.001), stage (P < 0.001), surgery (P < 0.001), chemotherapy (P < 0.001)and radiotherapy (P < 0.001) significantly affected patients’ CSS, while age (P = 0.378), sex (P = 0.055), race (P =0.831), household income (P = 0.234), marital status (P = 0.585) were not significant factors affecting CSS. (The results are also visualized in **Figure 4**). Further multivariate analysis using Cox proportional hazards model showed that patients with lymph node metastasis (HR = 1.842, 95%CI: 1.046-3.242) had worse CSS. Patients with regional extent (HR = 1.303, 95% CI: 0.506-3.356) and distant extent (HR = 4.956, 95% CI: 2.074-11.845) had shorter CSS relative to localized extent. Compared with non-surgical patients, surgery could appropriately prolong CSS (PP: HR = 0.015, 95% CI: 0.045-0.294; PD: HR = 0.271, 95% CI: 0.130-0.565; TP: HR = 0.552, 95%CI: 0.211-1.443) (**Figure 5**).

**Figure 6:**
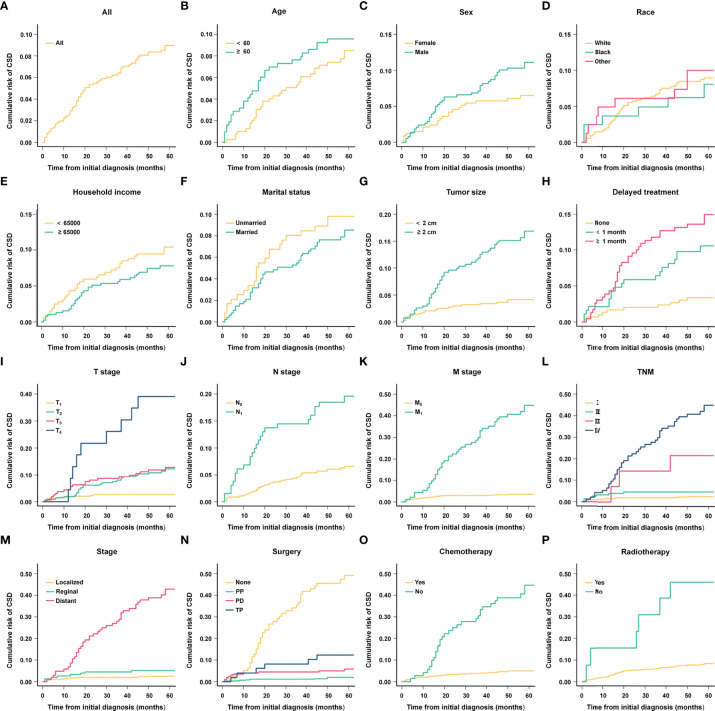
Cumulative risk curves of CSD in different subgroups. **(A)** All; **(B)** Age; **(C)** Sex; **(D)** Race; **(E)** Household income; **(F)** Marital status; **(G)** Tumor size; **(H)** Delayed treatment; **(I)** T stage; **(J)** N stage; **(K)** M stage; **(L)** TNM; **(M)** Stage; **(N)** Surgery; **(O)** Chemotherapy; **(P)** Radiotherapy.

**Figure 7:**
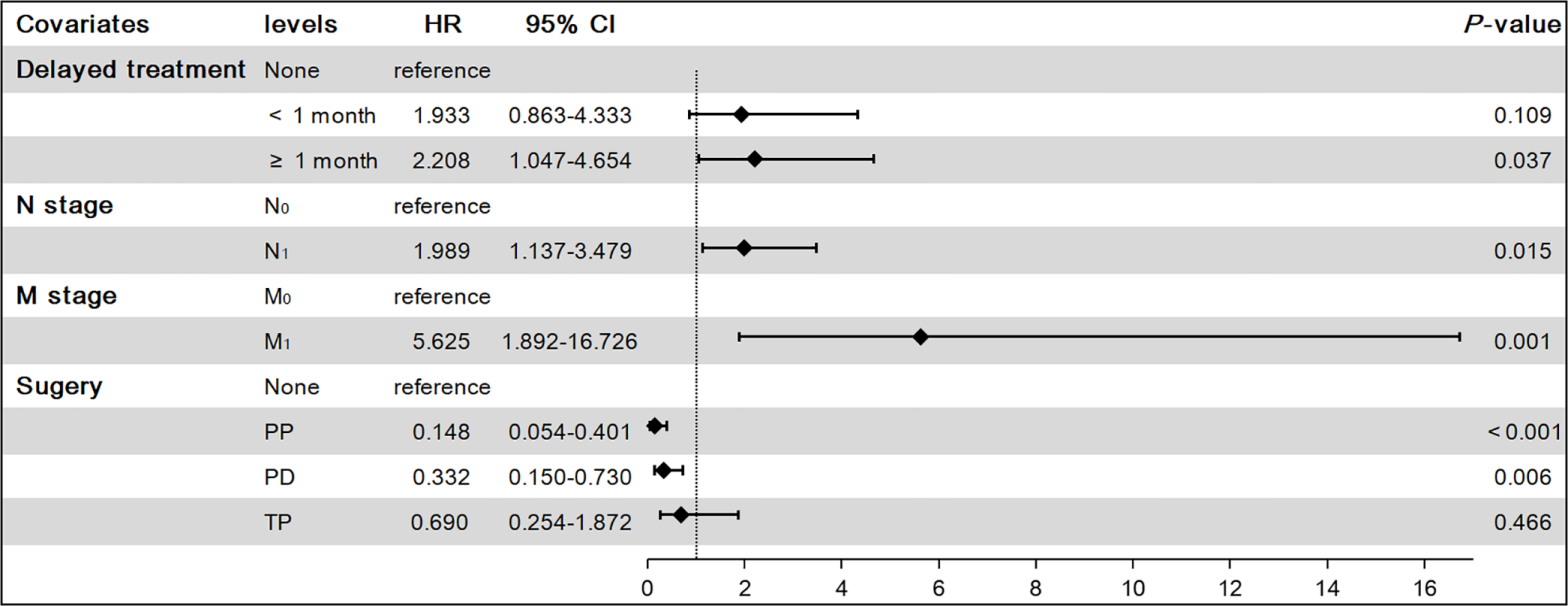
Results of multivariate competing-risk model on cancer-specific survival (CSS). PP, Partial pancreatectomy; PD, Pancreatectomy and duodenectomy; TP, Total pancreatectomy.

Using the above analysis method will overestimate the risk of CSD occurrence, resulting in competing risk bias. Therefore, we regarded death caused by other causes as a competing event and established a competing-risk model for analysis. The univariate analysis showed that age (*P* = 0.421), sex (*P* = 0.062), race (*P* = 0.828), household income (*P* = 0.226), and marital status (*P* = 0.572) were not significant factors affecting CSS. While tumor size (*P* < 0.001), delayed treatment (*P* < 0.001), TNM (*P* < 0.001), stage (*P* < 0.001), surgery (*P* < 0.001), radiotherapy (*P* < 0.001) and chemotherapy (*P* < 0.001) significantly affected patients’ CSS (**Table 2**). The 1-year, 3-year and 5-year cumulative incidences of CSD in patients with G1 NETs were 2.51%, 6.47% and 8.96%, respectively. In addition, the cumulative incidence of CSD in different subgroups is shown in **Table 3** and **Figure 6**. Then, to explore independent influencing factors for CSS, we performed a multivariate analysis of variables with *P-*values < 0.1 from univariate analysis. The results showed that delayed treatment, N stage, M stage, and surgery were independent influencing factors for CSS (**Figure 7**). Patients with G1 NETs whose treatment was delayed after diagnosis had a higher risk of developing CSD (delayed treatment < 1 month: HR = 1.933, 95%CI:0.863-4.333; delayed treatment ≥ 1 month: HR = 2.208, 95% CI: 1.047-4.654). Patients with lymph node metastasis(HR = 1.989, 95% CI: 1.137-3.479) or distant metastasis(HR = 5.625, 95% CI: 1.892-16.726) had a higher risk of developing CSD. Compared with non-surgical patients, surgery could reduce the incidence of CSD (PP: HR = 0.148, 95% CI: 0.0054-0.401; PD: HR = 0.332, 95% CI: 0.150-0.730; TP: HR = 0.69, 95% CI: 0.254-1.872).

**Table 2:** Univariate competitive risk analysis of G1 pNETs CSS.

*P* value			
		CSD	Other cause
Age	< 60	0.421	0.002
	≥ 60		
Sex	Female	0.062	0.051
	Male		
Race	White	0.828	0.902
	Black		
	Other		
Household income	< 65000	0.226	0.675
	≥ 65000		
Marital status	Married	0.572	0.043
	Unmarried		
Tumor size	< 2cm	<0.001	0.717
	≥ 2cm		
Delayed treatment	None	<0.001	0.986
	< 1 month		
	≥ 1 month		
T stage	T1	<0.001	0.340
	T2		
	T3		
	T4		
N stage	N0	<0.001	0.656
	N1		
M stage	M0	<0.001	0.197
	M1		
TNM	I	<0.001	0.222
	II		
	III		
	IV		
Stage	Localized	<0.001	0.155
	Regional		
	Distant		
Surgery	None	<0.001	0.400
	PP		
	PD		
	TP		
Radiotherapy	No	<0.001	0.533
	Yes		
Chemotherapy	No	<0.001	0.887
	Yes		

PP, Partial pancreatectomy; PD, Pancreatectomy and duodenectomy; TP, Total pancreatectomy; CSS, cancer-specific survival; CSD, cancer-specific death; G1 pNETs, grade 1 Pancreatic neuroendocrine tumors.

**Table 3:** Cumulative incidence of CSS in 1, 3 and 5 year among different subgroups for G1 pNETs survivors.

Covariant	Subgroup	n (%)	1 year (%)	3 year (%)	5 year (%)
Age	< 60	412 (54.9)	1.25	5.56	8.49
	≥ 60	339 (45.1)	4.11	7.62	9.58
Sex	Female	405 (53.9)	2.09	5.74	6.47
	Male	346 (46.1)	2.89	7.11	11.10
Race	White	580 (77.2)	1.99	6.75	8.95
	Black	85 (11.3)	3.66	4.90	8.05
	Other	86 (11.5)	4.88	6.11	9.99
Household income	< 65000	335 (44.6)	3.73	7.51	10.38
	≥ 65000	416 (55.4)	1.52	5.61	7.79
Marital status	Married	501 (66.7)	2.09	5.69	9.80
	Unmarried	250 (33.3)	3.35	8.02	8.55
Tumor size	< 2cm	464 (61.8)	2.02	3.17	4.13
	≥ 2cm	287 (38.2)	3.30	11.80	16.87
Delayed treatment	None	314 (41.8)	1.68	2.36	3.41
	< 1 month	194 (25.8)	2.15	6.46	10.58
	≥ 1 month	243 (32.4)	3.88	11.81	14.96
T stage	T1	326 (43.4)	1.90	2.55	2.55
	T2	230 (30.6)	1.84	7.93	12.18
	T3	172 (22.9)	4.32	9.26	12.76
	T4	23 (3.1)	4.34	26.08	39.13
N stage	N0	614 (81.8)	1.54	4.66	6.56
	N1	137 (18.2)	6.85	14.49	19.61
M stage	M0	654 (87.1)	1.93	2.90	3.41
	M1	97 (12.9)	6.37	29.81	44.92
TNM	I	478 (63.6)	1.53	1.97	2.44
	II	162 (21.6)	3.31	4.63	4.63
	III	14 (1.9)	0.01	14.28	21.42
	IV	97 (12.9)	0.06	29.81	44.92
Stage	Localized	478 (63.6)	1.53	1.97	2.44
	Regional	166 (22.1)	2.58	4.51	5.16
	Distant	107 (14.2)	6.72	28.90	42.93
Surgery	No	81 (10.8)	6.32	36.70	49.20
	PP	409 (54.5)	0.77	1.03	1.87
	PD	211 (28.1)	3.96	4.46	5.70
	TP	50 (6.7)	4.00	8.13	12.35
Radiotherapy	No	737 (98.1)	2.27	6.01	8.26
	Yes	14 (1.9)	15.38	30.76	46.15
Chemotherapy	No	676 (90.0)	2.17	3.74	4.88
	Yes	75 (10.0)	5.55	30.55	44.69

PP, Partial pancreatectomy; PD, Pancreatectomy and duodenectomy; TP, Total pancreatectomy; CSS, cancer-specific survival; G1 pNETs, grade 1 Pancreatic neuroendocrine tumors.

## 4. Discussion

The overall incidence of NENs has continued to increase over the past 40 years, and the incidence of pNETs has also increased (8–10). pNETs are heterogeneous tumors of the neuroendocrine system originating from islet cells of the pancreas (11). Most pNETs have relatively indolent behavior, but a small subset may exhibit a more aggressive phenotype (12, 13). Therefore, most clinical investigators focus on the study of G3 pNETs (14, 15). Compared with common pancreatic tumors, there is limited consensus on the clinicopathological features, treatment and prognosis of G1 pNETs. In this study, we used the SEER database to explore clinical features and factors associated with survival outcomes in patients with G1 NETs.

Classical survival analyses, including Kaplan-Meier analysis and Cox proportional hazards model, typically have only one study endpoint. The results of these analyses are reliable on OS. However, as for CSS, classical analytical methods would lead to biased results due to competing risk events. Therefore, our study used a competing-risk model to identify influencing factors for CSS in patients, as we considered not only deaths of G1 NETs but also deaths of other events, bringing the results closer to the truth.

Using the Cox proportional hazards model, we found that N stage, stage, and surgery independent influencing factors for CSS. Differently, we found that delayed treatment, N stage, M stage, and surgery were the main influencing factors for CSS by using the competing-risk model analysis. Compared with the competing-risk model, we found that the Cox model may have some bias in estimating the association of independent risk factors with outcomes. Ultimately, the competing-risk model has more tremendous advantages in accurately identifying factors affecting CSS.

G1 pNETs can occur at any age, and our study found that patients younger than 60 had better OS, which is consistent with previous findings (16). G1 pNETs are potentially malignant despite their slow growth and indolent clinical presentation. Due to the late onset of clinical symptoms in patients, the time to definite diagnosis is delayed and the incidence of distant metastasis is high (12.9% in this study), hindering the long-term survival of many patients (17, 18). Therefore, it is crucial for the early identification of G1 pNETs. Furthermore, our study confirmed that G1 pNETs have a better prognosis if detected and treated early. In the stage, we observed that patients with distant extent had the worst OS. As for lymph node or distant metastases, Izumo W et al. found that the number and extent of lymph node metastases were inversely correlated with OS in patients (19). It can be concluded that early detection of G1 pNETs is important. In addition, our study also found that patients treated immediately after initial diagnosis of G1 pNETs had a better prognosis, and delayed treatment after diagnosis, especially for more than one month, was associated with a significantly higher risk of CSD. These results suggest that it is advisable for early treatment after weighing factors such as age, symptoms, and tumor stage.

Our results showed that tumor size was also associated with OS and CSS in patients. The incidence of pNETs smaller than 2 cm has become increasingly common over the past few years, and there is debate over the optimal treatment (surgery or observation) for tumors smaller than 2 cm. Some consensus recommendations suggest that non-functioning tumors no larger than 2 cm should be considered for observation (20, 21). In 2022, Yang Z et al. conducted a study on pNETs smaller than 2 cm and found that the OS of the surgery group was all due to the observation group (22). In our study, pNENs less than 2 cm had a 96.9% surgical rate. Ultimately, the decision on whether to recommend resection of pNETs will be based on various factors, including tumor extent, anatomical location, tumor growth, tumor grade, proliferation markers (Ki-67), and presence of symptoms. For patients with localized pNETs, radical surgery is a curative treatment. Multiple studies have demonstrated that surgical resection prolongs survival in G1 pNETs (23, 24). Our study also found that the surgical group had better survival than the non-surgical group, which is consistent with previous findings (9, 25, 26). The majority of patients received surgical treatment for PP. Gratian et al. (27) found that compared with other surgical modalities, patients with pNETs undergoing PD had a poorer prognosis, which may be related to more severe preoperative obstructive jaundice, higher surgical risk, and lower postoperative quality of life in patients received PD. Age should also be considered when choosing surgical treatment because younger patients are generally better tolerated with pancreatic surgery complications and are expected to live longer. However, some patients are diagnosed at an advanced stage or with metastases that cannot be treated by surgical resection (3). For patients who cannot undergo radical surgery, treatment goals are to prolong survival, improve and maintain quality of life, and control tumor growth. In recent years, systemic treatment options have gradually increased, and biological therapy, chemotherapy, and radiopharmaceutical therapy have become the main treatment methods for patients with G1 and G2 pNETs (28–31).

For advanced and well-differentiated (G1, G2) pNETs, targeted therapy and chemotherapy are still critical therapeutic approaches. So far, only two targeted drugs have been approved for NETs: the tyrosine kinase inhibitor, Sunitinib (32, 33) and the mammalian target of rapamycin (mTOR) inhibitor, Everolimus (34, 35). Both improve progression-free survival in patients with advanced pNETs (32, 34, 35). Chemotherapy options include temozolomide with or without capecitabine (23), but for pNETs, especially with distant metastases, the efficacy is not as good as targeted therapy. Peptide receptor radionuclide therapy (PRRT) is recommended when the disease progresses after targeted therapy or chemotherapy or when there is no significant improvement in clinical symptoms (31, 36–38). ^177^Lu-PRRT can achieve long-term survival in patients with locally advanced or oligometastatic pNETs (39, 40). However, we found that chemotherapy failed to prolong survival in pNETs and radiotherapy was a risk factor for OS, possibly related to the low number of patients receiving radiotherapy. These do not demonstrate the benefit of radiotherapy for patients with G1 pNETs, as the specific regimens of radiotherapy and chemotherapy for pNETs are not documented in the SEER database. Therefore, the prognostic value of radiotherapy and chemotherapy for G1 pNETs has not been fully validated.

These suggest that surgery-based comprehensive treatment is the best way to achieve a good long-term prognosis for patients with G1 pNETs. The formulation of the surgical strategy should comprehensively consider the patient’s systemic status, tumor function and biological characteristics and carefully evaluate the risks and benefits of surgery.

This study is the first to use the SEER database to evaluate the influencing factors of OS and CSS in G1 pNETs patients. Compared with Cox proportional hazards model, the competing-risk model is more accurate in estimating the influencing factors of CSS. However, there are still some limitations to our study. First, as a retrospective study, bias is unavoidable due to incomplete information, such as a lack of information on the comorbidities of patients. Second, the small number of patients who received chemotherapy or radiotherapy in the SEER database and the lack of detailed information on chemotherapy and radiotherapy made it difficult for us to conduct further research. Finally, most NETs can be biotherapeutic with somatostatin analogues (SSA) due to overexpression of the somatostatin receptor (41, 42), but the SEER database does not record this information, which requires our further study.

## 5 Conclusion

In conclusion, by using the national-scale SEER database for research analysis, this study emphasizes the importance of early diagnosis and timely treatment of G1 pNETs. At present, surgical resection is still the main treatment for G1 pNETs, and further research is needed on the survival benefits of chemotherapy and radiotherapy. Comprehensive management of G1 pNETs may require multicenter collaboration to improve our understanding of this rare tumor and identify optimal treatment strategies to maximize the long-term patient quality of life.

## Data availability statement

The raw data supporting the conclusions of this article will be made available by the authors, without undue reservation.

## Ethics statement

Ethical review and approval was not required for the study on human participants in accordance with the local legislation and institutional requirements. Written informed consent for participation was not required for this study in accordance with the national legislation and the institutional requirements.

## Author contributions

ZW and XQ contributed to the conception and design of the study, ZW, XQ and YZ collected the details of the patients and extracted and analyzed the data. ZW and XQ drafted the manuscript. GL and XS contributed with a critical revision of the manuscript. All authors contributed to the article and approved the submitted version.

## Funding

This study was supported by the Jilin Province Department of Science and Technology, Grant/Award Number: 20200603001SF.

## Conflict of interest

The authors declare that the research was conducted in the absence of any commercial or financial relationships that could be construed as a potential conflict of interest.

## Publisher’s note

All claims expressed in this article are solely those of the authors and do not necessarily represent those of their affiliated organizations, or those of the publisher, the editors and the reviewers. Any product that may be evaluated in this article, or claim that may be made by its manufacturer, is not guaranteed or endorsed by the publisher.
